# A Hypothesis-Driven, Near-Peer Physical Diagnosis Module on Streptococcal Pharyngitis Within the Pediatrics Clerkship

**DOI:** 10.15766/mep_2374-8265.11448

**Published:** 2024-10-04

**Authors:** Lindsay Podraza, Lauren S. Starnes, Kyle Langford, Logan Garfield, Allyson Metro, Alyssa Schlotman, Nicole Chambers, Maya Neeley

**Affiliations:** 1 Third-Year Resident, Department of Pediatrics, Monroe Carell Jr. Children's Hospital at Vanderbilt; 2 Hospital Medicine Fellow, Department of Pediatrics, Monroe Carell Jr. Children's Hospital at Vanderbilt; 3 Associate Professor, Department of Pediatrics, Monroe Carell Jr. Children's Hospital at Vanderbilt

**Keywords:** Physical Examination, Physical Diagnosis, Near-Peer Learning, Residents as Teachers, Pediatrics, Clinical Skills Assessment/OSCEs, Clinical Teaching/Bedside Teaching, Clinical/Procedural Skills Training

## Abstract

**Introduction:**

In busy clinical settings, there is limited time to teach physical examination (PE) and procedural skills, particularly when the traditional head-to-toe PE approach is time-consuming. Near-peer teaching of a more efficient approach, the hypothesis-driven PE (HDPE), increases students’ learning opportunities. We developed a near-peer HDPE module to improve medical student confidence, knowledge, and skills for diagnosing and managing streptococcal pharyngitis.

**Methods:**

During this 1-hour module, residents taught the diagnostic approach for a patient with sore throat and facilitated small groups for practicing PE and throat swab skills. We assessed students using pre- and postmodule surveys including Likert-scale confidence scores (1 = *not at all confident*, 5= *extremely confident*), multiple-choice knowledge questions, and a skills rubric. A control group was surveyed at clerkship conclusion.

**Results:**

Of the 71 pediatric clerkship students who participated, 69 (97%) completed premodule surveys and 65 (91%) completed skills assessments. Twenty-eight (39%) completed postmodule surveys and skill assessments. After participation, students’ survey responses and rubrics indicated significant increase in confidence (*Mdn*_pre_ = 2 [IQR = 1,2], *Mdn*_post_ = 4 [IQR = 4,5]; *p* < .001), knowledge (*M*_pre_ = 40%, *M*_post_ = 77%; *p* < .001), and skills (*M*_pre_ = 5.3, *M*_post_ = 7.5; *p* < .01). Participating students also had significantly higher confidence (*p* < .005) and knowledge (*p* < 0.01) compared to the control group.

**Discussion:**

This near-peer HDPE module improved students’ knowledge, confidence, and skills related to streptococcal pharyngitis diagnosis and management and achieved compliance for a required clerkship skill.

## Educational Objectives

By the end of this activity, learners will be able to:

1. Apply modified Centor score criteria to stratify patients into risk categories for determining need for diagnostic testing/antibiotic treatment.
2. Describe the role of antibiotics in treatment of streptococcal pharyngitis.
3. Identify the most sensitive and specific physical examination findings of streptococcal pharyngitis.
4. Demonstrate physical examination skills relevant to a chief complaint of sore throat.
5. Perform a diagnostic throat swab procedure with proper technique.

## Introduction

Pediatric clerkships differ across the country in length and content. However, students should learn history-taking, physical examination, work-up, and procedural skills relevant to the most common pediatric illnesses. In the United States, sore throat is the sixth most common reason for an outpatient visit, and streptococcal pharyngitis causes 30% of pediatric cases.^1–3^ Given its common occurrence in a pediatric setting, we felt that learning physical diagnosis skills related to a patient with sore throat was important to incorporate within the clerkship, including how to effectively examine and perform a diagnostic throat swab on a patient. The throat swab skill cannot always be easily taught within the clerkship, as this is often performed in the outpatient setting or emergency department where students have minimal presence.

Several studies have demonstrated an ongoing decline in physical diagnosis skills among medical students and physicians, often attributed to less time spent at the bedside and more reliance on advanced diagnostic technologies.^4–6^ Studies have also shown that a diagnosis-specific physical diagnosis curriculum can improve knowledge and self-confidence in physical examination skills, but studies are limited to adult patients and acute otitis media in pediatrics.^7,8^ We utilized a hypothesis-driven approach in building our physical diagnosis module, rather than the traditional head-to-toe approach to learning the physical examination. When utilizing the latter approach, students learn an abundance of maneuvers and may struggle in applying their findings to support or reject a unifying diagnosis.^9^ Furthermore, a fast-paced, high-volume academic medical setting prompts the need for a more efficient teaching strategy.^9^ The hypothesis-driven physical examination (HDPE) offers an effective way to teach medical students physical examination skills.^10^ Through the utilization of illness scripts, such as a patient presenting with a sore throat, students can learn to actively choose maneuvers, synthesize key examination findings, and use clinical reasoning to diagnose and treat common diseases more efficiently.^3^ An evidence-based model from the literature, such as the *Journal of the American Medical Association* Rational Clinical Examination series, can serve as a foundation for the development of a HDPE-based physical diagnosis curriculum.^3^ To our knowledge, this is the first study to incorporate the HDPE as a framework for teaching both the physical examination and a related procedural skill.

While the HDPE is an ideal framework to teach these skills, it still requires dedicated time on the part of the educator, which can be challenging due to increasing demands for faculty physicians. Thus, we used a near-peer small-group model with senior residents as teachers. This strategy has many benefits, including the fostering of a nonthreatening learning environment and increasing learning opportunities for medical students.^11^ There can also be benefits for the near-peer teacher, such as consolidating their own learning and developing a professional identity.^12^ Furthermore, studies suggest that small-group learning in medical education leads to increased student investment, satisfaction, and examination scores.^13^ Taken together, our study is novel in that it investigated use of the near-peer small-group model to teach the HDPE in conjunction with a procedural skill.

## Methods

The authors of this study consisted of senior pediatrics residents, a pediatric hospital medicine fellow, and the pediatric clerkship director at our institution, who is a practicing pediatric hospitalist. Using Kern's six-step approach to curriculum design, we started with a general needs and problem assessment, in addition to a literature review.^14^ We recognized that our institution's preclinical skills curriculum included limited pediatrics exposure and used the head-to-toe methodology in teaching the physical examination, rather than a hypothesis-driven approach. Students complete this 13-month course prior to starting clerkships. With lack of exposure to pediatrics-specific teaching, the pediatrics clerkship serves as the sole opportunity to hone these skills. However, as is common across many medical schools in the United States, the required amount of time students spend on pediatrics clerkships is less than other core clerkships, further limiting time for students to solidify their knowledge and skills.^15^ At our institution, each pediatrics clerkship cohort consists of 15–20 students. Students receive an orientation at the start of the 6-week pediatrics clerkship. During the clerkship, students are required to participate in a list of procedures, skills, and diagnoses relevant to pediatrics through immersive experiences. One required procedural skill is the throat swab. Through a targeted needs assessment, we found that students were most frequently missing the throat swab experience and decided to target this for our module. Prior to our module, there was no formal method to achieve total compliance or assess mastery of procedural skills.

We designed a near-peer, HDPE module with the goal of assessing the effectiveness of this teaching strategy on improving medical student confidence, knowledge, and skills related to diagnosing and treating streptococcal pharyngitis in pediatric patients. We mapped desired outcomes to Kirkpatrick's first three levels including reaction, learning, and behavior.^16^

To connect teaching a procedural skill to physical diagnosis, we took a hypothesis-driven approach similar to Fagan et al.^7^ Using the article “Does This Patient Have Strep Throat?”^3^ as a framework, we highlighted the most specific physical examination findings, clinical prediction rules, diagnostic strategies, and treatment for streptococcal pharyngitis. We used Fitts and Posner's automaticity and skill expertise conceptual framework, which emphasizes the cognitive, associative, and autonomous phases of skills acquisition.^17^

Prior to the first session, senior residents attended a meeting to review proper technique of the examination maneuvers and throat swab technique. They then led a 1-hour required orientation session that included four educational components: (1) a baseline practical skills (physical examination and throat swab technique) assessment, (2) didactic session, (3) demonstration of a sore throat-focused physical examination and throat swab, and (4) hands-on practice. The 20-minute didactic consisted of a PowerPoint presentation on streptococcal pharyngitis (Appendix A), including topics such as patient presentation, sensitive and specific physical examination findings, Centor score criteria with McIsaac modification, diagnostic testing, treatment, and case-based scenarios.^2,3,18,19^ We included representative photos for key examination findings (i.e., palatal petechiae, tonsillar enlargement/exudate, and scarlatiniform rash). Then, students broke into small groups for the remainder of the hour to practice the physical examination and throat swab procedure. Within each group, a resident demonstrated how to accurately perform a focused examination and throat swab (see Facilitator Guide, Appendix B). One-by-one, students then practiced the examination and throat swab on each other while the resident provided real-time feedback on their technique.

Each student was then encouraged, but not required, to attend a second 1-hour session later in the clerkship which was offered several afternoons throughout each block to optimize student participation and allow for smaller student-teacher ratios. At the start of this session, residents assessed students’ practical skills. Afterwards, students participated in resident-led bedside teaching rounds on admitted patients, with a specific emphasis on the focused physical examination, using a checklist (Appendix C).

### Assessment Strategies

We developed two instruments to assess our module. We utilized an anonymous REDCap survey to assess knowledge and confidence (Appendices D and E) before and after participation in the module.^20^ The pre- and postmodule knowledge assessment included five multiple-choice questions covering topics from the didactic session such as Centor-scoring a patient in a clinical vignette, treatment strategy, specific/sensitive examination findings, and appropriate throat swab technique. The confidence assessment included 5-point Likert-scale questions (1 = *not confident,* 5 = *very confident*) to assess confidence in diagnosing, testing, and treating streptococcal pharyngitis. The postmodule survey (Appendix E) also included a question on perceived benefit from the module and a free-text qualitative feedback question on suggestions for improvement, which two authors inductively coded for themes.

To evaluate practical skills (i.e., physical examination and throat swab technique), we created a Throat Swab Skills Assessment Rubric (Appendix F) that included twelve focused physical examination items and procedure-related tasks relevant to assessing a patient presenting with a sore throat. Two authors (Lindsay Podraza and Lauren S. Starnes) independently reviewed the *Journal of the American Medical Association* article^3^ and Elsevier evidence-based throat specimen collection checklist^21^ for potential physical examination and procedure-related tasks relevant to assessing a patient presenting with a sore throat for inclusion in the rubric. The authors discussed the examination skills and tasks until an agreement was reached on which were most important for rubric inclusion. If a consensus could not be reached, a third author (Maya Neeley) who was the pediatrics clerkship director, decided if the item should be included.

Residents evaluated students with the Throat Swab Skills Assessment Rubric twice: once at the beginning of their clerkship orientation and again at the beginning of the second session of this module. During the assessment, students paired up (one serving as the physician and the other as the patient) and a resident prompted them to perform a focused physical examination and throat swab on a patient presenting with a sore throat and verbalize their findings. Students were given 2 minutes to perform the task. We provided all required materials (i.e., gloves, tongue depressors, and throat swabs). Residents observed each student perform the task and completed the rubric (Appendix F). Resident evaluators were not necessarily consistent between sessions due to scheduling conflicts, but they were coached to execute the assessment in a standardized way to reduce evaluator-to-evaluator variability.

Clerkship students from the first block of the academic year did not experience the module and served as our control group. They completed the postmodule survey (Appendix E) assessing knowledge and confidence at the conclusion of their clerkship. As there had not yet been time built into their clerkship for a skills assessment, our proxy to compare their skills to those of the intervention group was to compare the results of the knowledge question that required students to properly identify the correct sequence of procedural steps and technique in performing a throat swab (Appendix D, Knowledge Question 4).

### Statistical Analysis

Students who participated in the module during blocks 2 through 5 of the clerkship, were considered the intervention group. We compared the intervention group's premodule survey and practical skills rubric results to their postmodule results. We also compared the intervention group's postmodule survey results to the control group's survey results using *t*-tests for knowledge and skills domains and Mann–Whitney *U* tests for the confidence domain.

Additionally, we compared knowledge and confidence of clerkship blocks 2 and 5 to assess for potential differences with academic year progression.

## Results

Our control group consisted of 22 students who had recently completed the pediatrics clerkship without module exposure (Block 1). Of those, 8 students (36%) responded to the postmodule survey.

Over the remainder of the academic year (Blocks 2–5), 71 students completed the clerkship with module incorporated, serving as the intervention group. Sixty-nine students (97%) completed the premodule survey and 28 students (39%) completed the postmodule survey.

### Confidence and Knowledge Survey

After participation in the module, students’ postmodule survey responses showed a significant increase in confidence and knowledge compared their premodule survey responses. The median confidence score increased from 2 (IQR = 1,2) to 4 (IQR = 4,5; *p* < .001; Table 1) on a 5-point Likert scale. The average knowledge score increased from 40% (*SD* = 20%) to 75% (*SD* = 19%; *p* < .001; Table 2). Postmodule survey responses, at the end of the clerkship, indicated that students who completed the module had significantly higher median confidence (*Mdn* = 4 [IQR = 4,5]) than the control group (*Mdn* = 3 [IQR = 3,4]; *p* < 0.005; Table 1), and significantly higher knowledge scores (*M* = 75% [*SD* = 19%]) compared to the control group (*M* = 50% [*SD* = 21%]; *p* < 0.01) compared to the control group (Table 2).

**Table 1.**
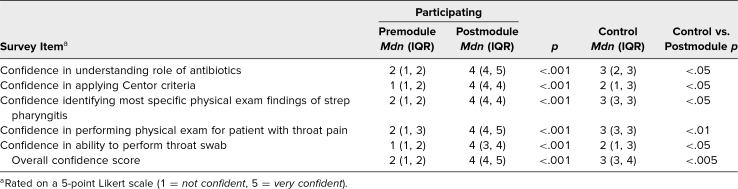
Confidence Scores for Participating (*N*_pre_ = 69, *N*_post_ = 28) and Nonparticipating (Control; *N* = 8) Students

**Table 2.**
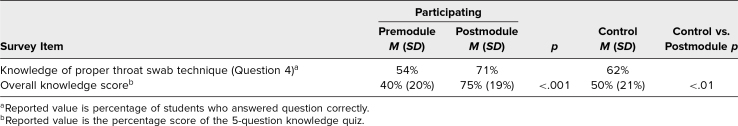
Knowledge Scores for Participating (*N*_pre_ = 69, *N*_post_ = 28) and Nonparticipating (Control; *N* = 8) Students

When analyzing the results of the knowledge question on properly identifying the correct sequence of procedural steps and throat swab technique (Appendix D, Question 4), we found that 62% of control student group students answered it correctly. In our intervention group, 54% of students answered it correctly before the module, compared to 71% of students after the module (Table 2).

There was no significance difference in knowledge or confidence scores of students in blocks 2 and 5. The average premodule knowledge score of block 2 and block 5 students was 43% (*SD* = 19%) and 54% (*SD* = 12%), respectively. The average postmodule score of block 2 and block 5 students was 73% (*SD* = 17%) and 67% (*SD* = 22%), respectively. The median confidence score for block 2 students was 1 (IQR = 1,2) on the premodule survey and 4 (IQR = 4,4) on the postmodule survey. The median confidence score for block 5 students was 2 (IQR = 1,2) on the premodule survey and 4 (IQR = 3,4) on the postmodule survey.

### Throat Swab Skills Rubric

Sixty-five (91%) students completed the premodule practical skills assessments and 28 students (39%) completed the postmodule skills assessments. Completed rubrics showed that the average of satisfactorily-performed skills significantly increased from 5.3 (*SD* = 1.7) to 7.6 (*SD* = 1.5; *p* < .001) out of a total of 12 skills.

### Student Evaluation of Module

We identified four major themes in qualitative feedback: appreciation for near-peer teaching, learning through repetitive practice, novelty of content, and preparedness for patient care. Students commented on the benefits of near-peer teaching, especially observing residents model behaviors and giving feedback. One student wrote, “Seeing the residents perform a throat swab as they talked through the process was very helpful.” Another wrote, “I appreciated the multiple opportunities to practice the exam and the feedback that I received”. Students also commented on the unique exposure to the skill through the module and how it prepared them for clinical experiences. Students commented, “It was a great lesson to have before outpatient clinic,” “Having the chance to learn these techniques beforehand made me confident to perform the throat swab and gather the right clinical data when I was on outpatient clinic the next month,” “I was prepared to be able to swab the first patient that I saw with signs of having strep pharyngitis,” and “Having the teaching session really prepared me for learning on the wards.” 100% of students who responded reported that they benefited from the module.

## Discussion

Overall, we found that a near-peer, literature-based, HDPE module can improve medical student knowledge, confidence, and skills related to a diagnosis of streptococcal pharyngitis. We utilized the HDPE to build context around a required procedural skill that is commonly performed in a pediatric setting. As not every pediatric patient with a sore throat requires a diagnostic throat swab, our module emphasized using clinical reasoning to guide diagnostic decision-making. Students not only learned how to perform the procedure correctly, but also identified which patients should undergo testing based on clinical prediction rules and physical examination findings.

When comparing the intervention group's knowledge and confidence to the control group, we found that the control group's end-of-clerkship scores were higher than the intervention group's premodule scores but less than the intervention group's postmodule scores. While the control group results may demonstrate natural learning during the clerkship, we hypothesize that students experienced an added benefit with our module given the intervention group's higher postmodule scores. In addition, we initially hypothesized that early clerkship groups inherently would have less knowledge and confidence than later groups, but we learned from comparing block 2 and 5 data that this is not necessarily the case.

Our module was effective in systematically achieving near total compliance for a required clerkship experience and providing students with valuable feedback so they could improve their procedural technique. Importantly, all postmodule survey respondents felt that they benefited from the module.

A unique aspect of our work was the utilization of a near-peer small-group model to teach the HDPE. This approach was beneficial for providing an effective approach and increasing students’ opportunities to hone skills. Evidence suggests clinical skills are best taught with a low student-to-teacher ratio, which can be difficult to achieve in a high-volume, fast-paced setting.^22^ Near-peers themselves can benefit, as participation satisfies several ACGME core competencies (medical knowledge, professionalism, practice-based learning and improvement, and interpersonal and communication skills).^23^ Studies also suggest that teaching clinical skills can improve the teacher's own skills.^24^ Through our qualitative data, we learned that students particularly enjoyed the resident involvement in our module. However, a potential challenge to this near-peer strategy is that it takes diligent planning and coordination of residents’ schedules, so we recommend recruiting a large group of facilitators. We addressed this by utilizing residents interested in teaching or those on elective rotations.

This strategy would be effective to teach the clinical approach to a variety of diagnoses and associated procedures. Our strategy can be tailored towards various levels of learners and specialties and easily implemented using near peers, few resources, and minimal time.

Our evaluation is limited by our single-year implementation and a small sample size for both the intervention and control groups. Due to the ethical dilemma of withholding an educational intervention from a group of students during each clerkship block, our control group consisted of students from the block scheduled prior to module finalization, which was the first block of the academic year. As these students had already completed their clerkship, we were unable to assess this group's skills. We also acknowledge the potential confounding factors introduced into this study because of the inherent nature of designing an educational intervention within a clerkship experience. We are unable to capture the unique experiences students may have during their clerkship that could contribute to an increase in their knowledge, confidence, and skills relevant to the evaluated diagnosis. While we acknowledge that performing a throat swab on an adult is vastly different than on a pediatric patient, we opted to not use pediatric patients for throat swab practice due to the potentially uncomfortable nature of the procedure and no benefit for the patient from participating. We did, however, encourage students to perform this procedure throughout their clerkship experiences. Recency bias could have played a role in the skills assessment results, as students who had the second session soon after orientation may have scored higher than those who had the session at the end of their clerkship. In addition, the second session was not required, leading to a smaller number of completed postmodule skills assessments. Students who participated in this second session and survey may have been more motivated to participate, leading to response bias. In the future, it would be helpful require the second session within the clerkship to allow for more postmodule results.

Future directions include incorporating the HDPE approach in teaching physical examination and procedural skills to learners in medicine. With this, students can learn to identify key aspects of the history that necessitate focused evaluation of certain organ systems, modeling how physical examinations are performed in real clinical practice.^10^ This practical approach increases the value and efficiency of the physical examination and can prevent unnecessary diagnostic testing.^10^

Furthermore, increasing the involvement of near-peers in the preclinical and clinical curriculum can increase pediatric-specific learning opportunities. A study investigating the incorporation of near-peer teachers to increase exposure to pediatrics patients and skills is currently underway at our institution. We would also be interested in studying the effects on the near-peer facilitators after participation in this type of module, specifically as it pertains to their physical examination and procedural skills.

## Appendices


Physical Diagnosis Streptococcal Pharyngitis.pptxFacilitator Guide.docxSore Throat Physical Exam Bedside Checklist.docxPremodule Survey.docxPostmodule Survey.docxThroat Swab Skills Assessment Rubric.docx
*All appendices are peer reviewed as integral parts of the Original Publication.*
