# Supplementary material for: A Hypothesis-Driven, Near-Peer Physical Diagnosis Module on Streptococcal Pharyngitis Within the Pediatrics Clerkship
Source: MedEdPORTAL. 2024 Oct 4;20:11448. doi: 10.15766/mep_2374-8265.11448 (PMC11450068; doi:10.15766/mep_2374-8265.11448)
Supplement: Supplementary file 1 — Physical Diagnosis Streptococcal Pharyngitis.pptxFacilitator Guide.docxSore Throat Physical Exam Bedside Checklist.docxPremodule Survey.docxPostmodule Survey.docxThroat Swab Skills Assessment Rubric.docx [file mep_2374-8265.11448-s001.zip › A. Physical Diagnosis Streptococcal Pharyngitis.pptx]

## Slide 1
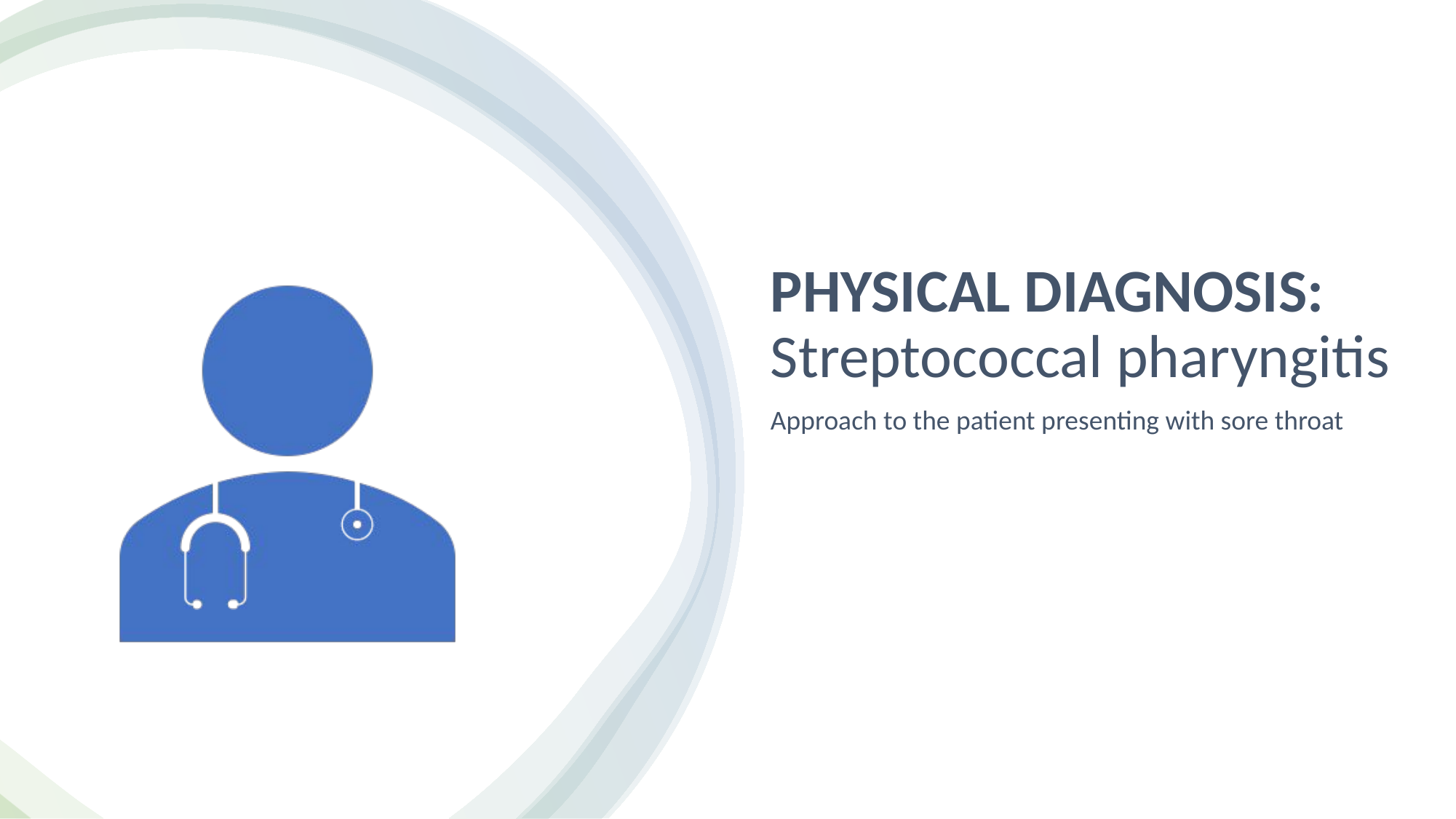

# PHYSICAL DIAGNOSIS: Streptococcal pharyngitis
Approach to the patient presenting with sore throat

## Slide 2
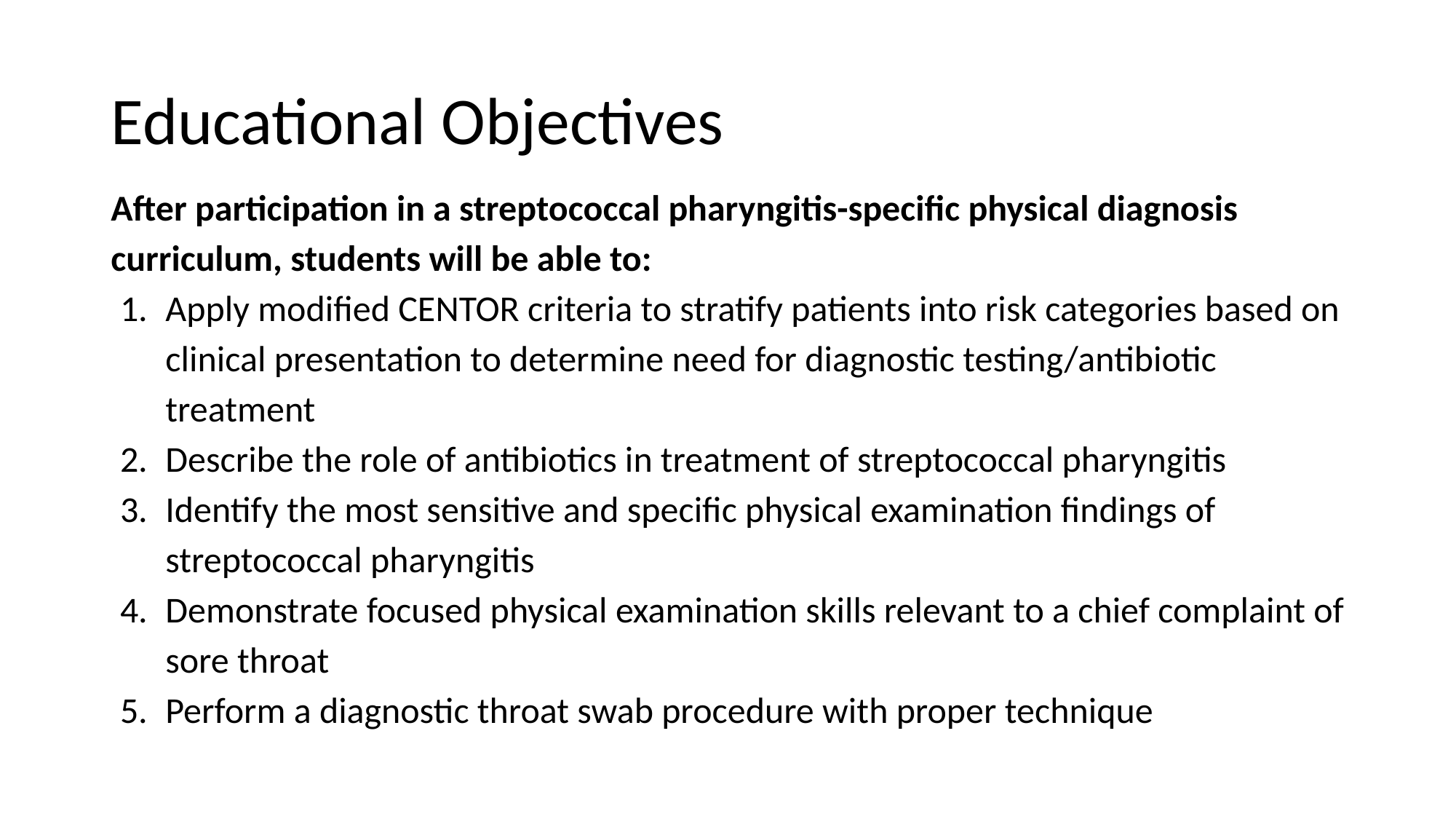

# Educational Objectives
After participation in a streptococcal pharyngitis-specific physical diagnosis curriculum, students will be able to:
Apply modified CENTOR criteria to stratify patients into risk categories based on clinical presentation to determine need for diagnostic testing/antibiotic treatment
Describe the role of antibiotics in treatment of streptococcal pharyngitis
Identify the most sensitive and specific physical examination findings of streptococcal pharyngitis
Demonstrate focused physical examination skills relevant to a chief complaint of sore throat
Perform a diagnostic throat swab procedure with proper technique

## Slide 3
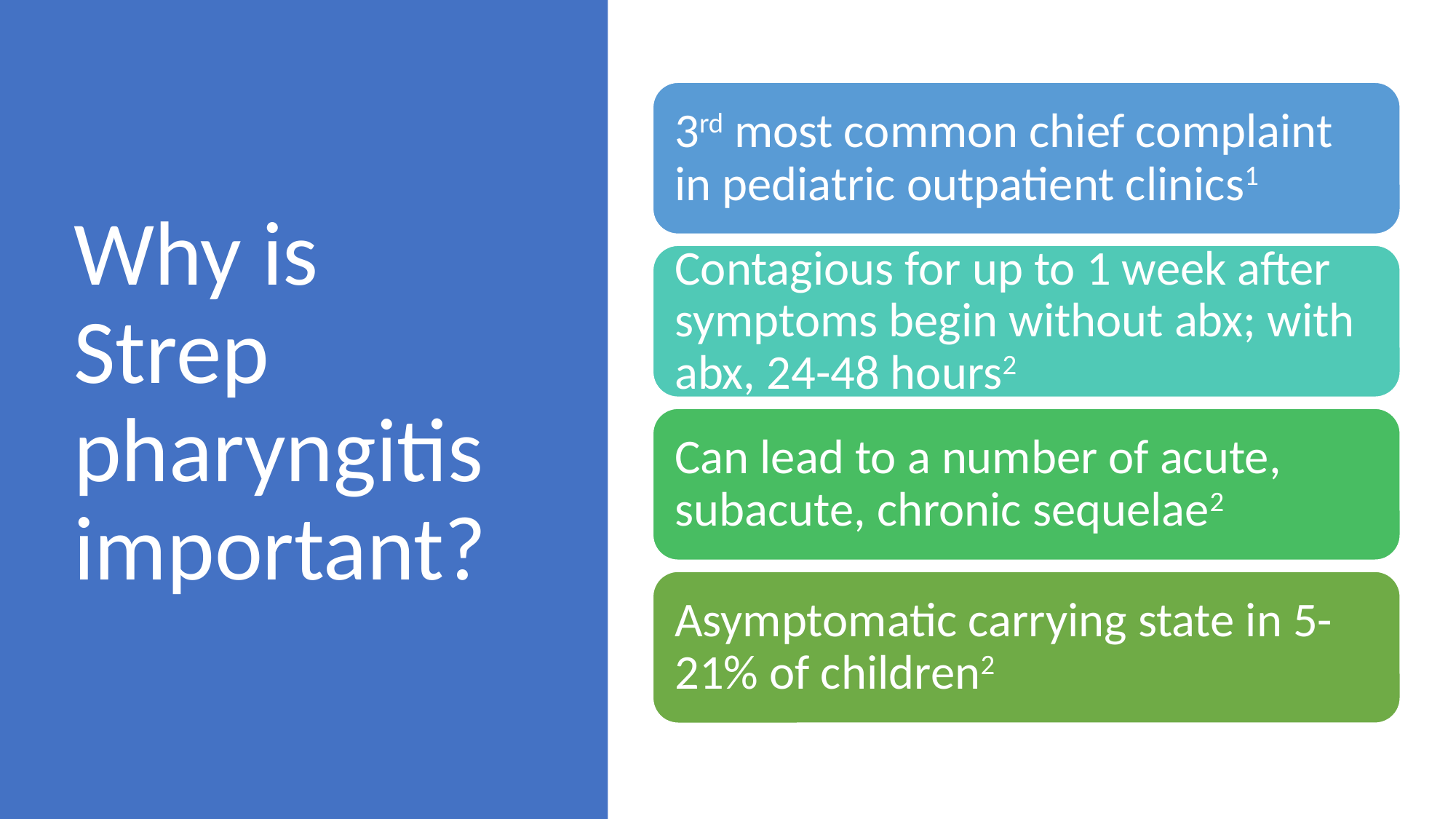

# Why is Strep pharyngitis important?
3rd most common chief complaint in pediatric outpatient clinics1
Contagious for up to 1 week after symptoms begin without abx; with abx, 24-48 hours2
Can lead to a number of acute, subacute, chronic sequelae2
Asymptomatic carrying state in 5-21% of children2

## Slide 4
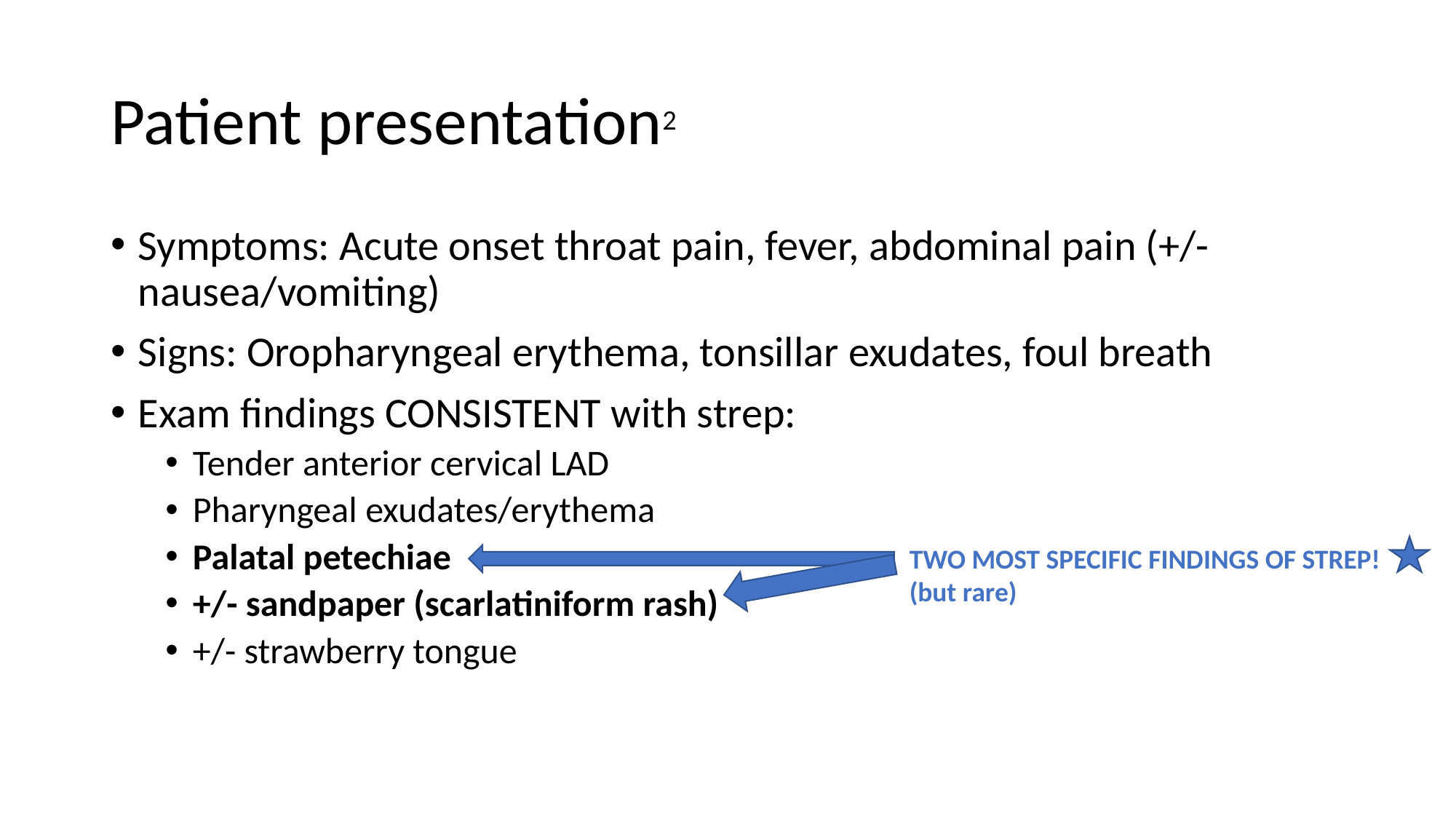

# Patient presentation2
Symptoms: Acute onset throat pain, fever, abdominal pain (+/- nausea/vomiting)
Signs: Oropharyngeal erythema, tonsillar exudates, foul breath
Exam findings CONSISTENT with strep:
Tender anterior cervical LAD
Pharyngeal exudates/erythema
Palatal petechiae
+/- sandpaper (scarlatiniform rash)
+/- strawberry tongue
TWO MOST SPECIFIC FINDINGS OF STREP!
(but rare)

## Slide 5
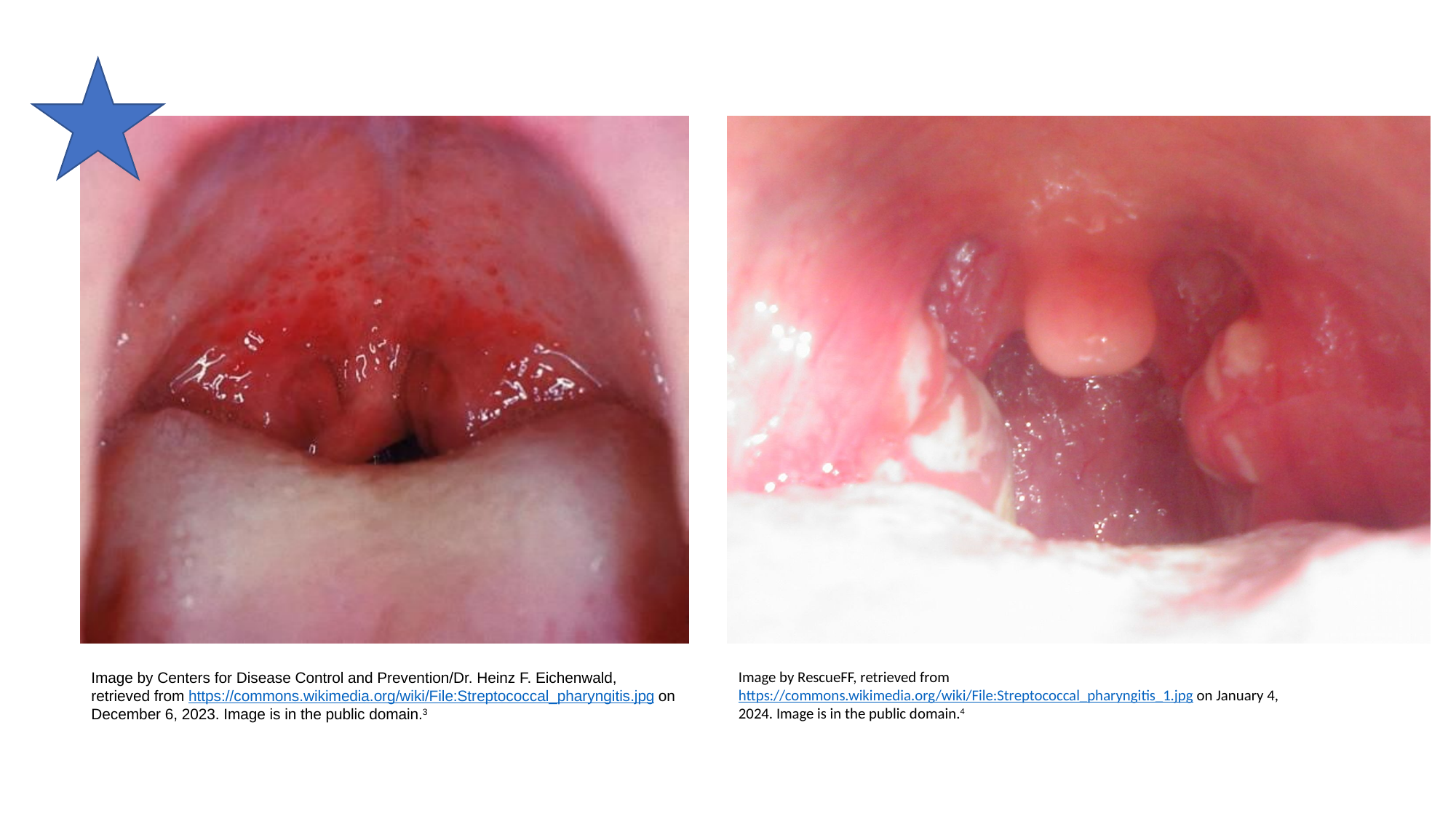

Image by RescueFF, retrieved from https://commons.wikimedia.org/wiki/File:Streptococcal_pharyngitis_1.jpg on January 4, 2024. Image is in the public domain.4
Image by Centers for Disease Control and Prevention/Dr. Heinz F. Eichenwald, retrieved from https://commons.wikimedia.org/wiki/File:Streptococcal_pharyngitis.jpg on December 6, 2023. Image is in the public domain.3

## Slide 6
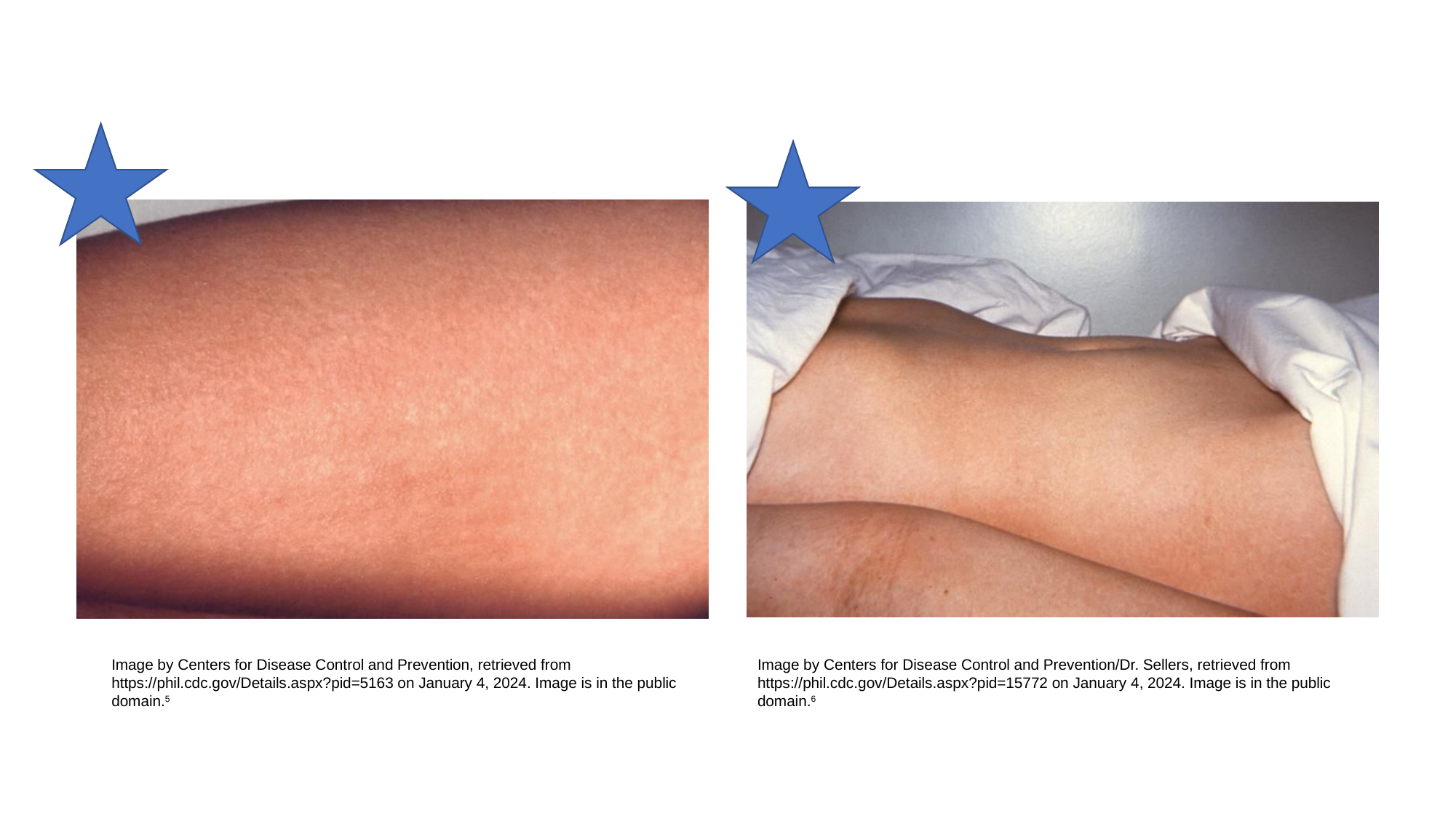

Image by Centers for Disease Control and Prevention, retrieved from https://phil.cdc.gov/Details.aspx?pid=5163 on January 4, 2024. Image is in the public domain.5
Image by Centers for Disease Control and Prevention/Dr. Sellers, retrieved from https://phil.cdc.gov/Details.aspx?pid=15772 on January 4, 2024. Image is in the public domain.6

## Slide 7
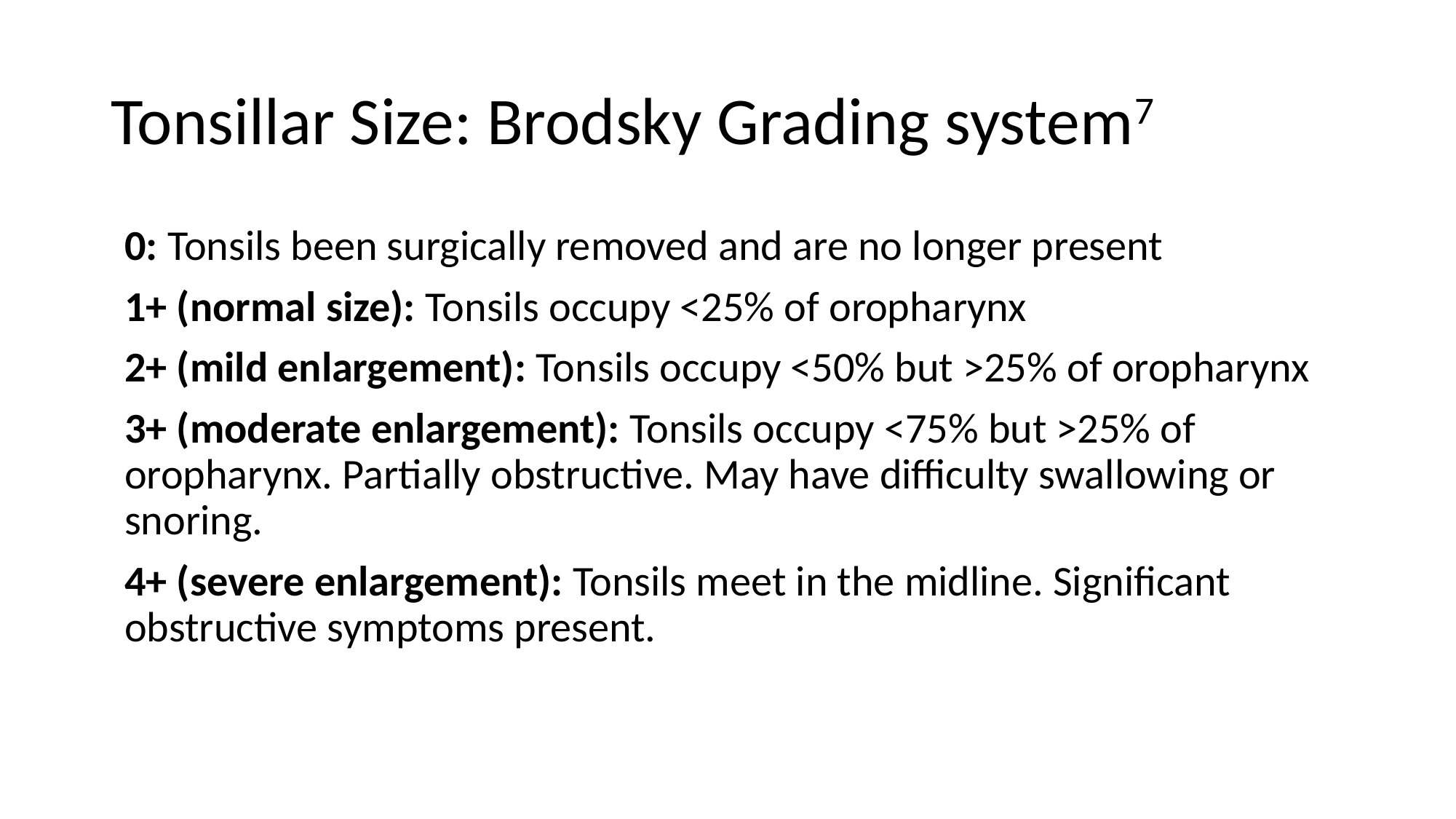

# Tonsillar Size: Brodsky Grading system7
0: Tonsils been surgically removed and are no longer present
1+ (normal size): Tonsils occupy <25% of oropharynx
2+ (mild enlargement): Tonsils occupy <50% but >25% of oropharynx
3+ (moderate enlargement): Tonsils occupy <75% but >25% of oropharynx. Partially obstructive. May have difficulty swallowing or snoring.
4+ (severe enlargement): Tonsils meet in the midline. Significant obstructive symptoms present.

## Slide 8
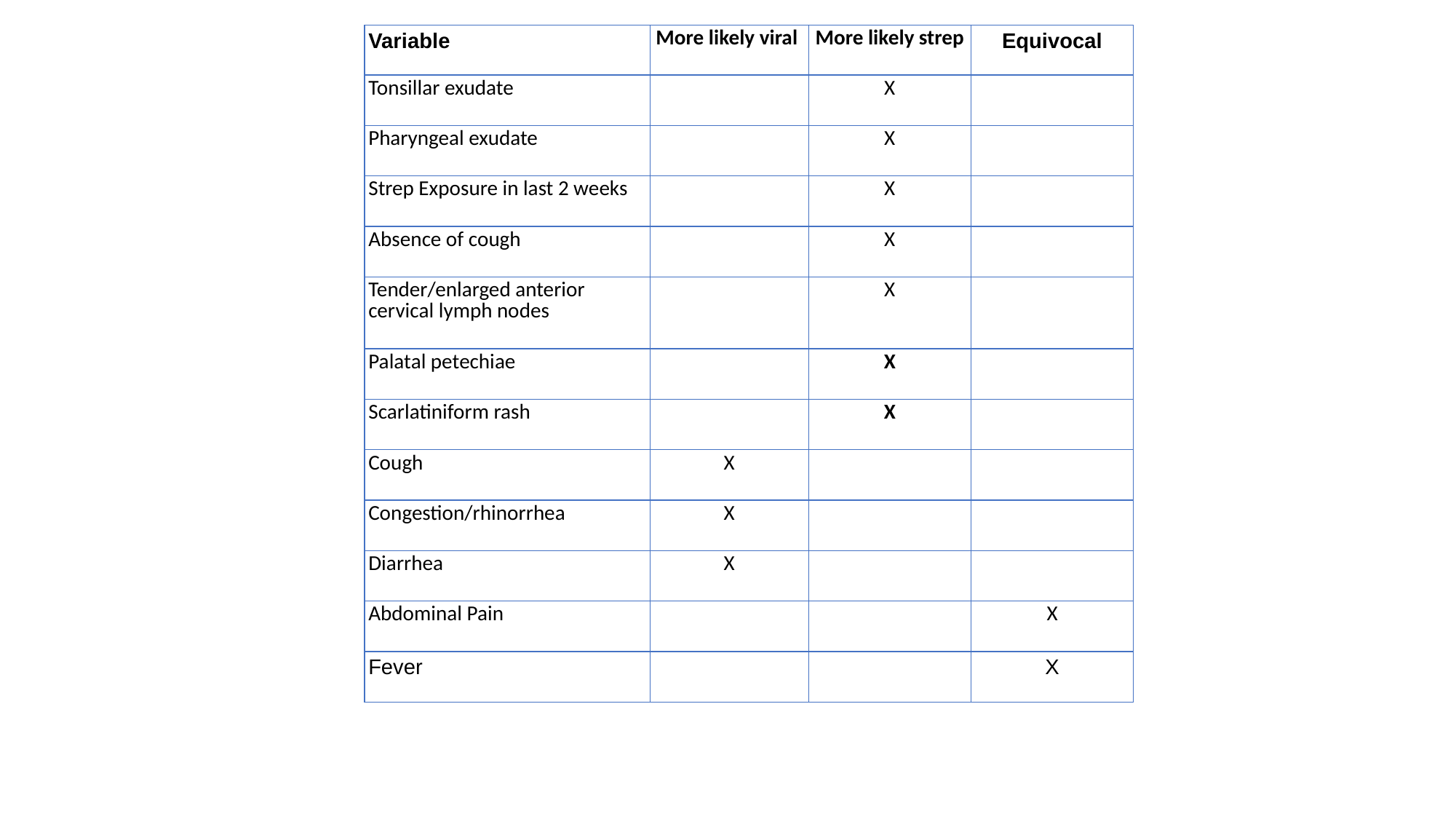

| Variable | More likely viral | More likely strep | Equivocal |
| --- | --- | --- | --- |
| Tonsillar exudate | | X | |
| Pharyngeal exudate | | X | |
| Strep Exposure in last 2 weeks | | X | |
| Absence of cough | | X | |
| Tender/enlarged anterior cervical lymph nodes | | X | |
| Palatal petechiae | | X | |
| Scarlatiniform rash | | X | |
| Cough | X | | |
| Congestion/rhinorrhea | X | | |
| Diarrhea | X | | |
| Abdominal Pain | | | X |
| Fever | | | X |

## Slide 9
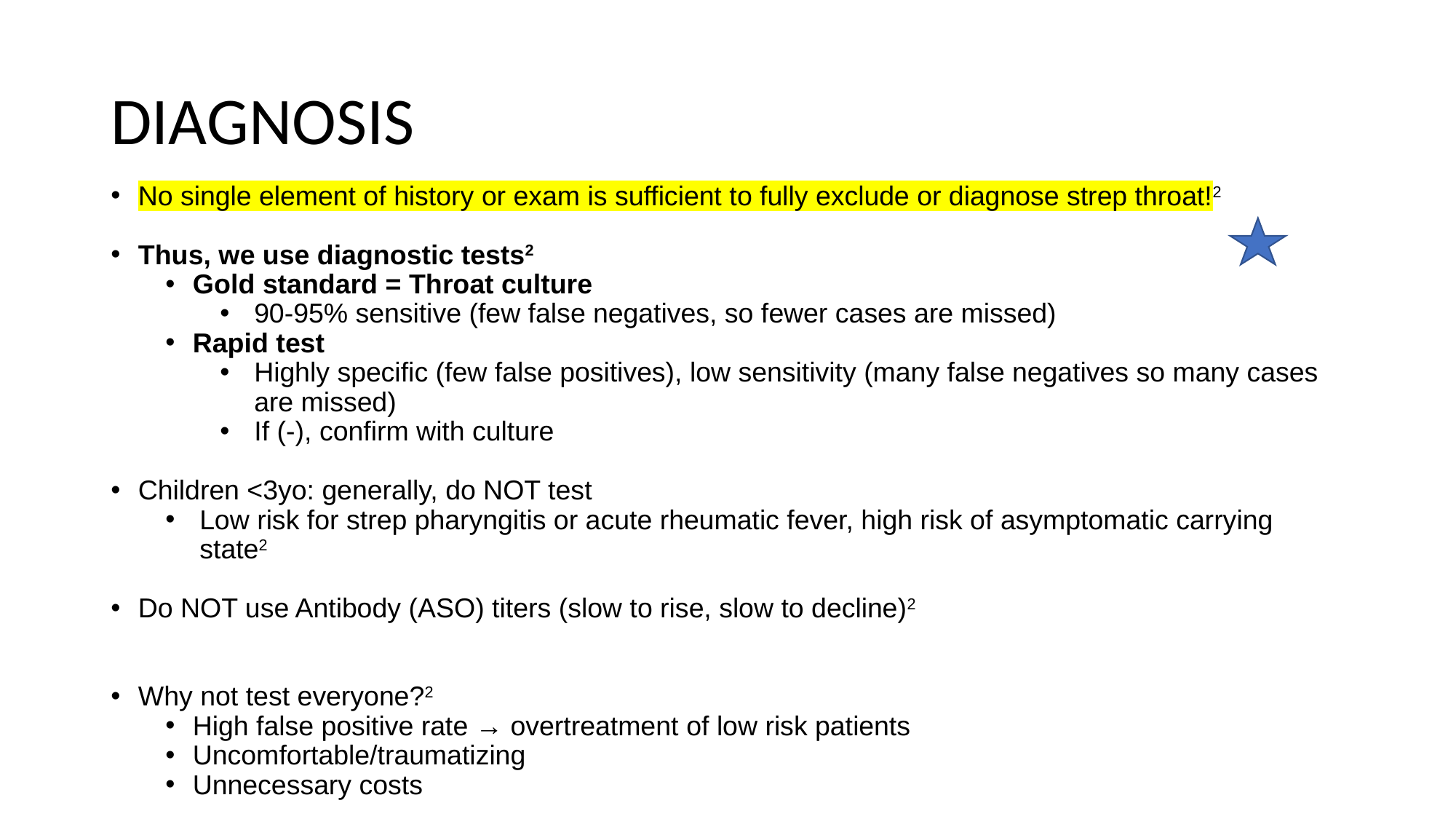

# DIAGNOSIS
No single element of history or exam is sufficient to fully exclude or diagnose strep throat!2
Thus, we use diagnostic tests2
Gold standard = Throat culture
90-95% sensitive (few false negatives, so fewer cases are missed)
Rapid test
Highly specific (few false positives), low sensitivity (many false negatives so many cases are missed)
If (-), confirm with culture
Children <3yo: generally, do NOT test
Low risk for strep pharyngitis or acute rheumatic fever, high risk of asymptomatic carrying state2
Do NOT use Antibody (ASO) titers (slow to rise, slow to decline)2
Why not test everyone?2
High false positive rate → overtreatment of low risk patients
Uncomfortable/traumatizing
Unnecessary costs

## Slide 10
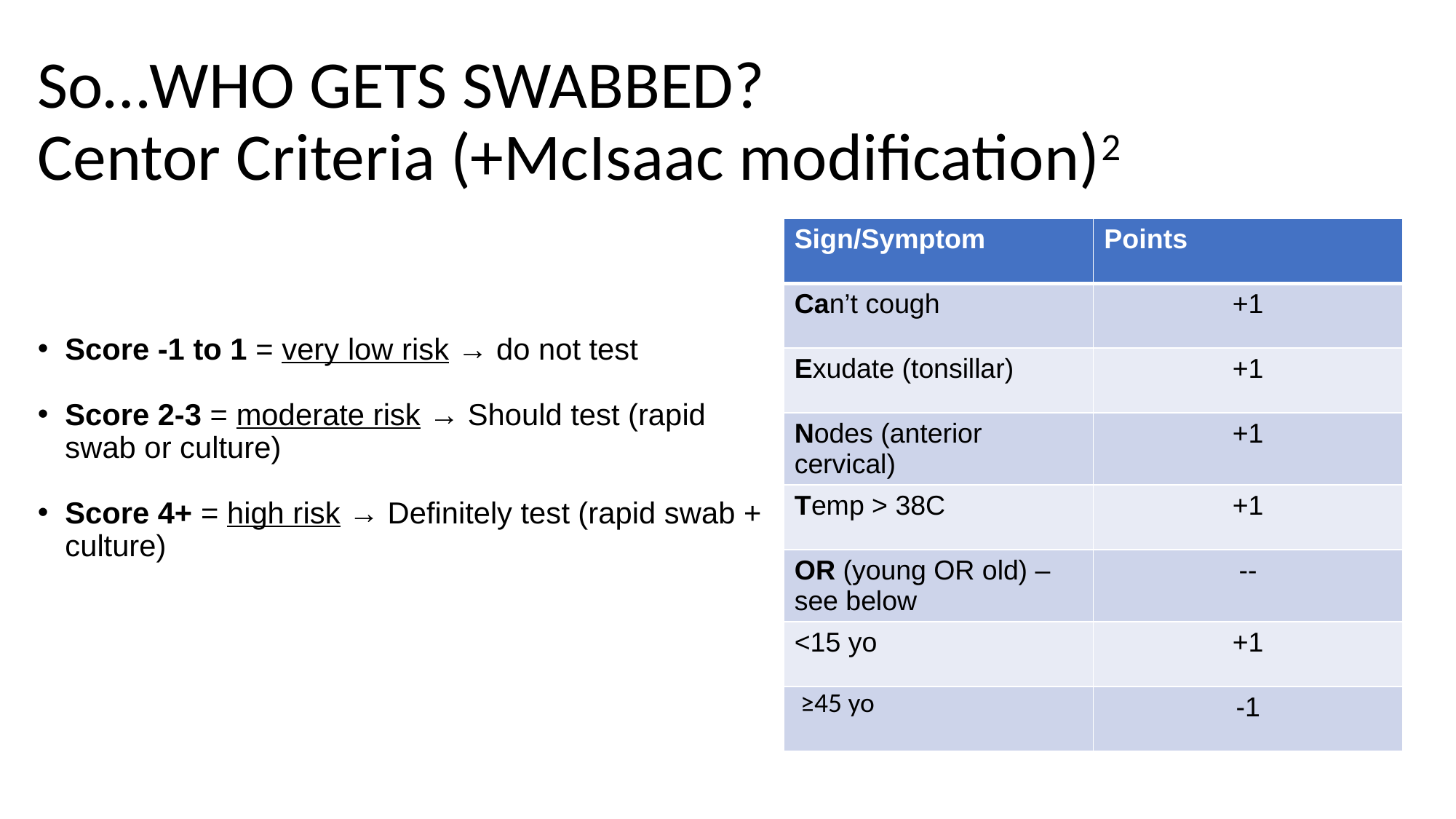

# So…WHO GETS SWABBED? Centor Criteria (+McIsaac modification)2
| Sign/Symptom | Points |
| --- | --- |
| Can’t cough | +1 |
| Exudate (tonsillar) | +1 |
| Nodes (anterior cervical) | +1 |
| Temp > 38C | +1 |
| OR (young OR old) – see below | -- |
| <15 yo | +1 |
| ≥45 yo | -1 |
Score -1 to 1 = very low risk → do not test
Score 2-3 = moderate risk → Should test (rapid swab or culture)
Score 4+ = high risk → Definitely test (rapid swab + culture)

## Slide 11
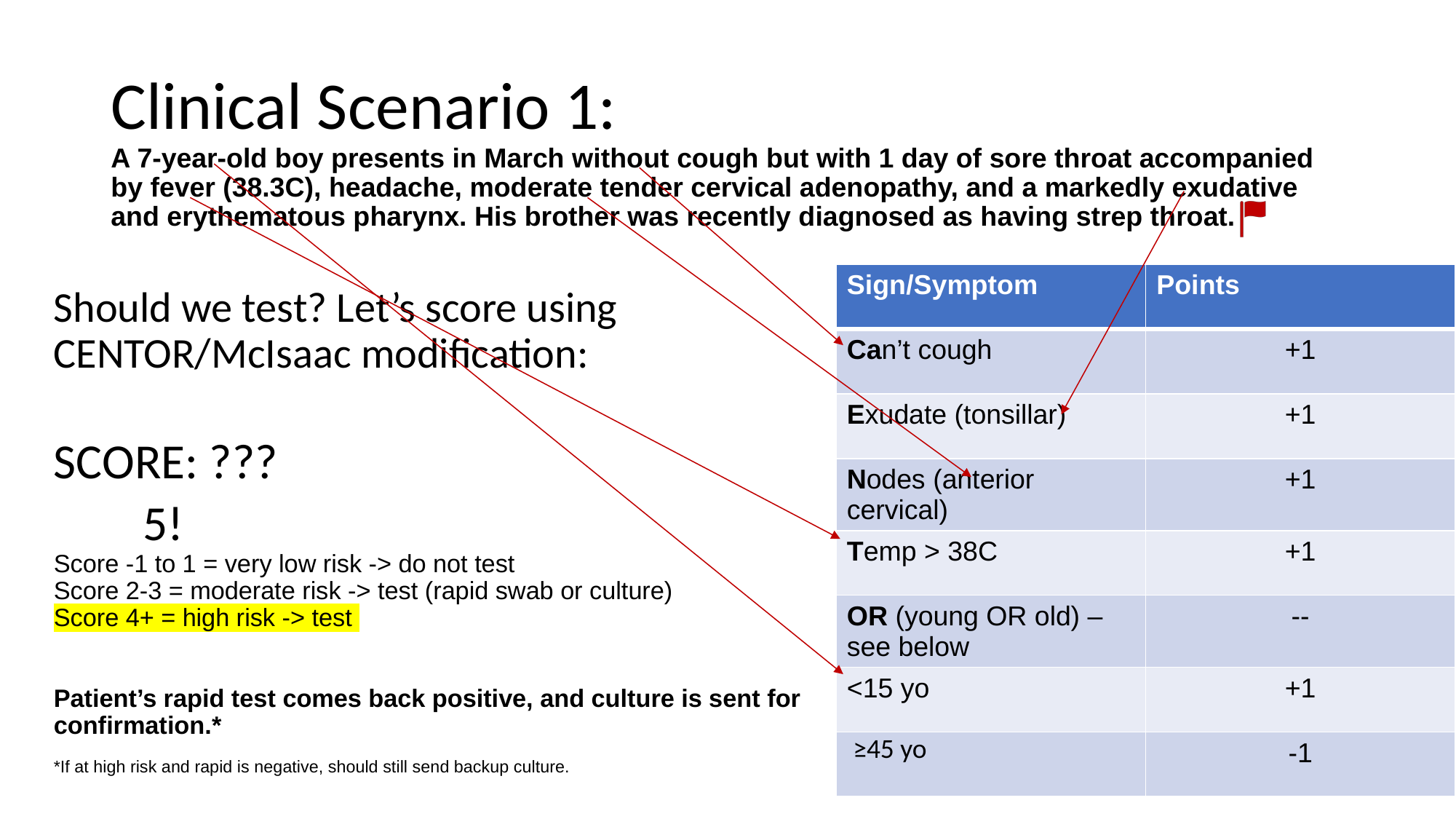

# Clinical Scenario 1: A 7-year-old boy presents in March without cough but with 1 day of sore throat accompanied by fever (38.3C), headache, moderate tender cervical adenopathy, and a markedly exudative and erythematous pharynx. His brother was recently diagnosed as having strep throat.
| Sign/Symptom | Points |
| --- | --- |
| Can’t cough | +1 |
| Exudate (tonsillar) | +1 |
| Nodes (anterior cervical) | +1 |
| Temp > 38C | +1 |
| OR (young OR old) – see below | -- |
| <15 yo | +1 |
| ≥45 yo | -1 |
Should we test? Let’s score using CENTOR/McIsaac modification:
SCORE: ???
 5!
Score -1 to 1 = very low risk -> do not test
Score 2-3 = moderate risk -> test (rapid swab or culture)
Score 4+ = high risk -> test
Patient’s rapid test comes back positive, and culture is sent for confirmation.*
*If at high risk and rapid is negative, should still send backup culture.

## Slide 12
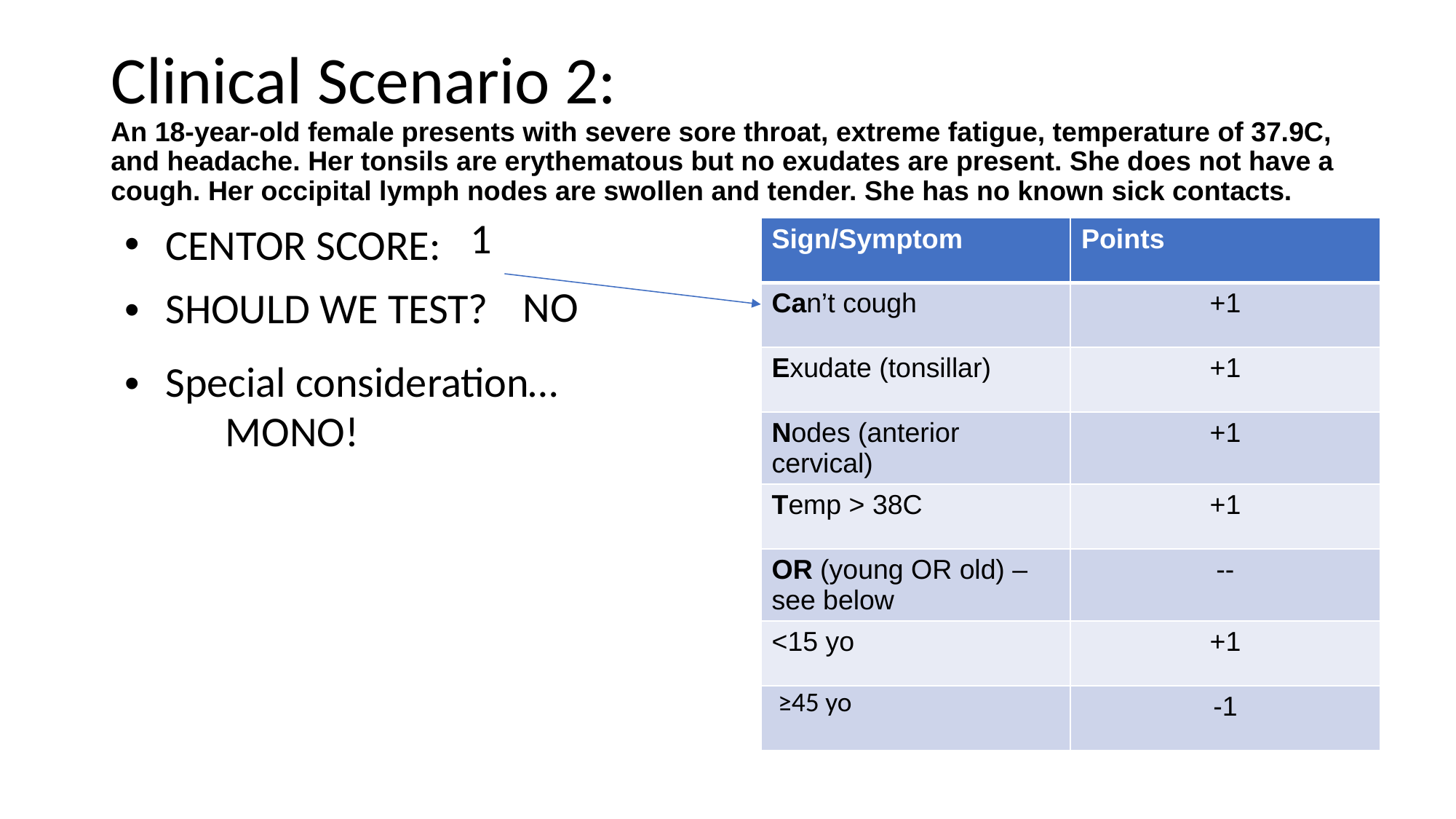

Clinical Scenario 2:
An 18-year-old female presents with severe sore throat, extreme fatigue, temperature of 37.9C, and headache. Her tonsils are erythematous but no exudates are present. She does not have a cough. Her occipital lymph nodes are swollen and tender. She has no known sick contacts.
1
CENTOR SCORE:
| Sign/Symptom | Points |
| --- | --- |
| Can’t cough | +1 |
| Exudate (tonsillar) | +1 |
| Nodes (anterior cervical) | +1 |
| Temp > 38C | +1 |
| OR (young OR old) – see below | -- |
| <15 yo | +1 |
| ≥45 yo | -1 |
NO
SHOULD WE TEST?
Special consideration…
MONO!

## Slide 13
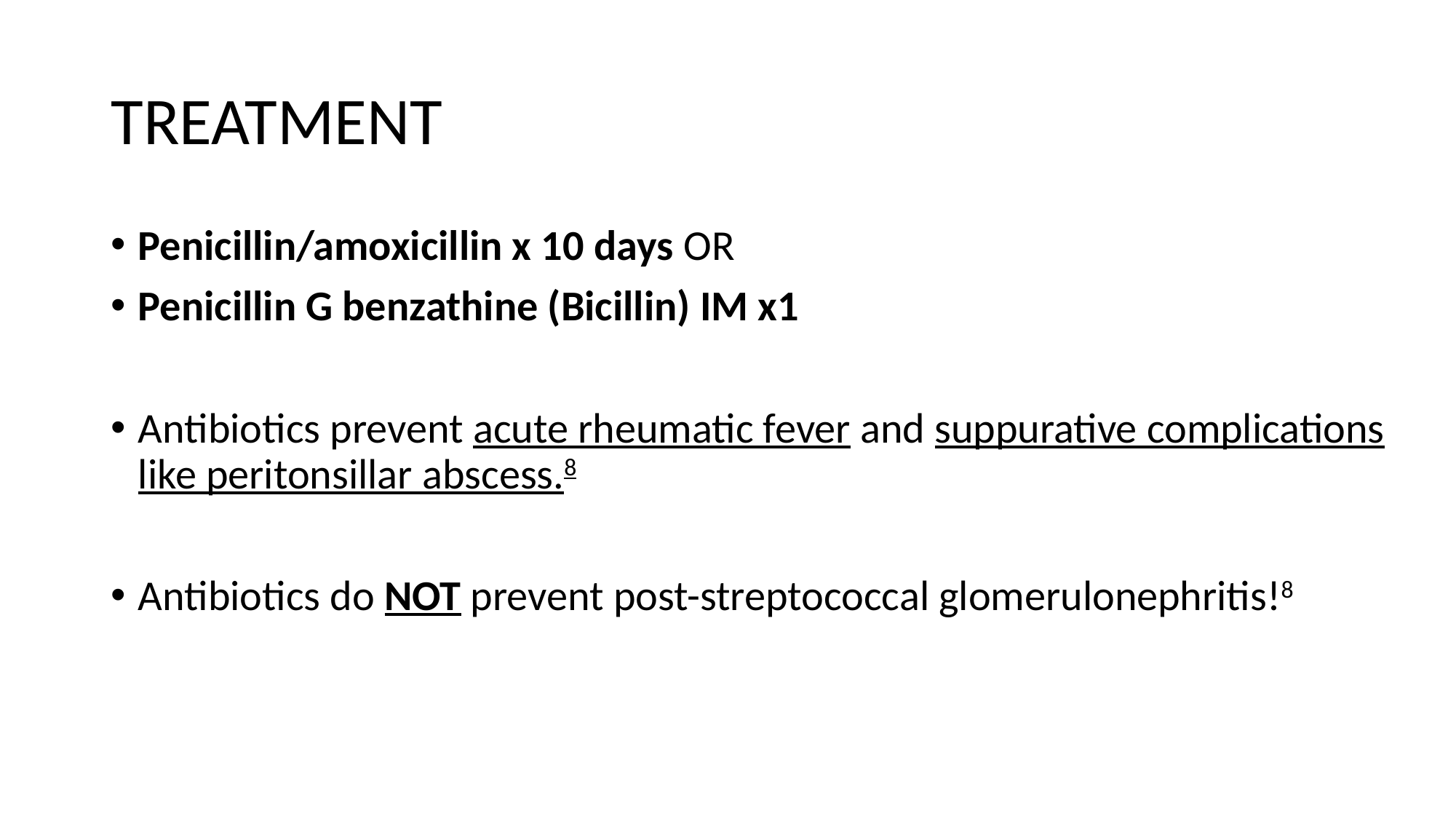

# TREATMENT
Penicillin/amoxicillin x 10 days OR
Penicillin G benzathine (Bicillin) IM x1
Antibiotics prevent acute rheumatic fever and suppurative complications like peritonsillar abscess.8
Antibiotics do NOT prevent post-streptococcal glomerulonephritis!8

## Slide 14
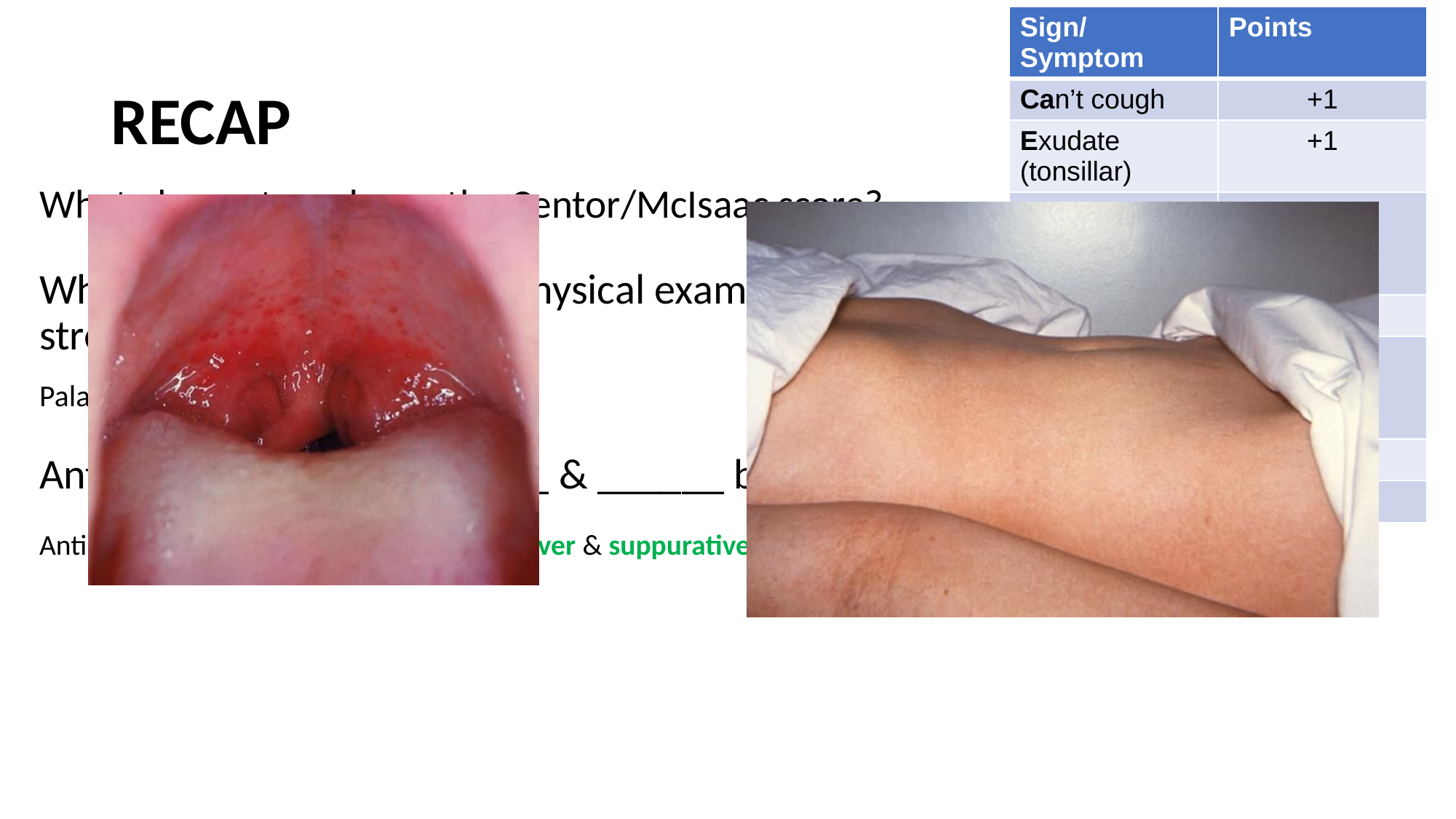

| Sign/Symptom | Points |
| --- | --- |
| Can’t cough | +1 |
| Exudate (tonsillar) | +1 |
| Nodes (anterior cervical) | +1 |
| Temp > 38C | +1 |
| OR (young OR old) – see below | -- |
| <15 yo | +1 |
| ≥45 yo | -1 |
# RECAP
What elements make up the Centor/McIsaac score?
What are the most specific physical exam findings of strep?
Palatal petechiae and scarlatiniform rash
Antibiotics can prevent _____ & ______ but NOT ______.
Antibiotics can prevent acute rheumatic fever & suppurative complications but NOT PSGN

## Slide 15
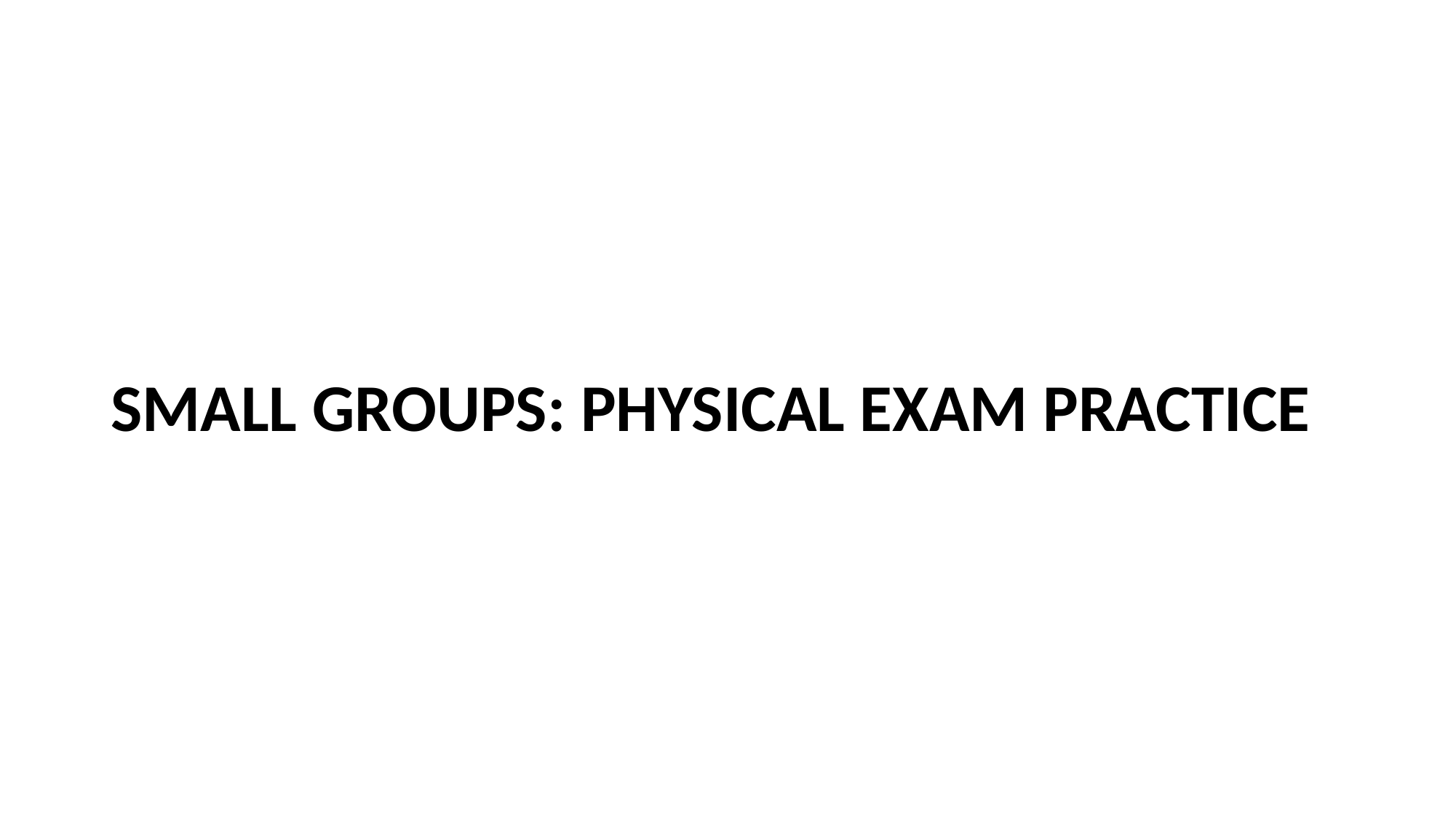

# SMALL GROUPS: PHYSICAL EXAM PRACTICE

## Slide 16
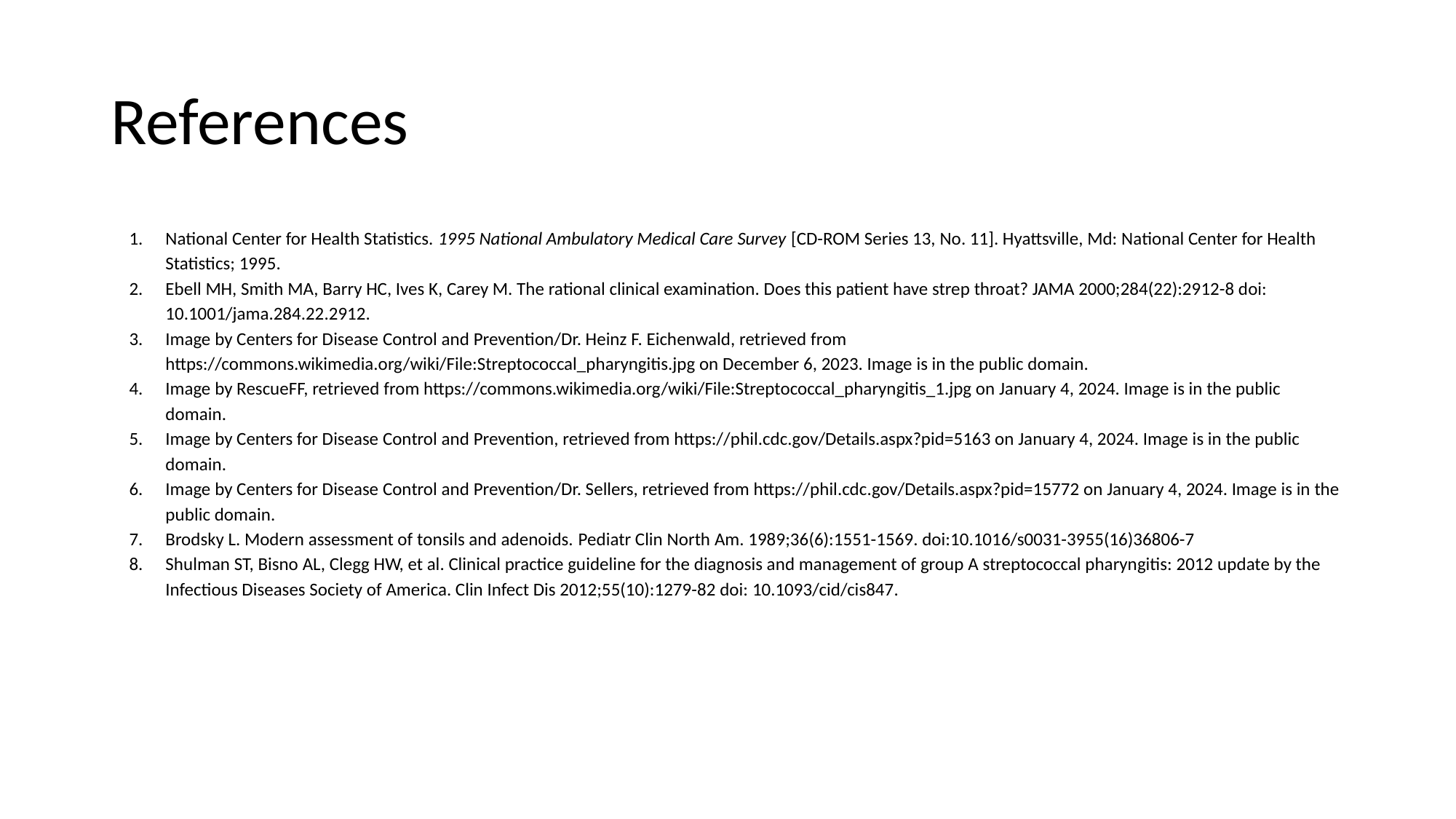

# References
National Center for Health Statistics. 1995 National Ambulatory Medical Care Survey [CD-ROM Series 13, No. 11]. Hyattsville, Md: National Center for Health Statistics; 1995.
Ebell MH, Smith MA, Barry HC, Ives K, Carey M. The rational clinical examination. Does this patient have strep throat? JAMA 2000;284(22):2912-8 doi: 10.1001/jama.284.22.2912.
Image by Centers for Disease Control and Prevention/Dr. Heinz F. Eichenwald, retrieved from https://commons.wikimedia.org/wiki/File:Streptococcal_pharyngitis.jpg on December 6, 2023. Image is in the public domain.
Image by RescueFF, retrieved from https://commons.wikimedia.org/wiki/File:Streptococcal_pharyngitis_1.jpg on January 4, 2024. Image is in the public domain.
Image by Centers for Disease Control and Prevention, retrieved from https://phil.cdc.gov/Details.aspx?pid=5163 on January 4, 2024. Image is in the public domain.
Image by Centers for Disease Control and Prevention/Dr. Sellers, retrieved from https://phil.cdc.gov/Details.aspx?pid=15772 on January 4, 2024. Image is in the public domain.
Brodsky L. Modern assessment of tonsils and adenoids. Pediatr Clin North Am. 1989;36(6):1551-1569. doi:10.1016/s0031-3955(16)36806-7
Shulman ST, Bisno AL, Clegg HW, et al. Clinical practice guideline for the diagnosis and management of group A streptococcal pharyngitis: 2012 update by the Infectious Diseases Society of America. Clin Infect Dis 2012;55(10):1279-82 doi: 10.1093/cid/cis847.
