# Supplementary material for: A Hypothesis-Driven, Near-Peer Physical Diagnosis Module on Streptococcal Pharyngitis Within the Pediatrics Clerkship
Source: MedEdPORTAL. 2024 Oct 4;20:11448. doi: 10.15766/mep_2374-8265.11448 (PMC11450068; doi:10.15766/mep_2374-8265.11448)
Supplement: Supplementary file 1 — Physical Diagnosis Streptococcal Pharyngitis.pptxFacilitator Guide.docxSore Throat Physical Exam Bedside Checklist.docxPremodule Survey.docxPostmodule Survey.docxThroat Swab Skills Assessment Rubric.docx [file mep_2374-8265.11448-s001.zip › B. Facilitator Guide.docx]

**Physical Diagnosis: Streptococcal Pharyngitis**

Facilitator Guide

**LARGE GROUP SESSION:**

**Materials required:**
- Gloves

- Tongue depressors

- Throat swabs

- Light source (may use phone if otoscopes unavailable)

**PART ONE: BASELINE SKILLS ASSESSMENT**

This session will begin with a baseline clinical skills (focused physical exam and throat swab skills) assessment. Instruct students to break up into small groups based on number of facilitators available. The goal is to get through all student assessments within 20 minutes. Students will be called up two at a time, ideally in a location away from the rest of the group. One student will be the physician, and the other will be the patient. You as the facilitator will be an observer. You will prompt the student doctor to “perform a focused physical exam on a patient presenting with sore throat and then perform a throat swab” and instruct them to verbalize what they are seeing (or not seeing) on exam. Of note, it is best if students rotate through the roles one-by-one rather than in pairs, as to avoid one student copying another. You as the facilitator will fill out the assessment rubric (Appendix F) while NOT providing any feedback or prompting. Each student should be allowed 2 minutes to complete the task.

**PART TWO: DIDACTIC**

Once all students have completed the baseline assessment, you will give a 25-minute didactic using the PowerPoint presentation (Appendix A). See the speaker notes in the comments.

**PART THREE: SMALL GROUP BREAKOUT SESSIONS**

You will have approximately **15 minutes** to complete this portion of the session.

Begin by asking a student (or fellow facilitator, if available) to be the patient. You will be the physician.

Demonstrate the focused physical examination as directed by the checklist below (important points/clarifications noted in parentheses). This should take approximately 7 minutes or less.

- HEENT:
  - Tonsils
    - Presence or absence (Has the patient had a tonsillectomy/adenoidectomy?)
    - Size (Brodsky Grading Scale 0-4+, see below)
      - 0: Tonsils been surgically removed and are no longer present
      - 1+ (normal size): Tonsils occupy <25% of oropharynx
      - 2+ (mild enlargement): Tonsils occupy <50% but >25% of oropharynx
      - 3+ (moderate enlargement): Tonsils occupy <75% but >25% of oropharynx. Partially obstructive. May have difficulty swallowing or snoring.
      - 4+ (severe enlargement): Tonsils meet in the midline. Significant obstructive symptoms present.
    - Color (Erythema = redness)
    - Exudates (Exudates = white pus)
  - Uvula
    - Deviated vs midline (deviated suggests deep neck infection)
    - Color
    - Petechiae? (Small reddish-purple spots)
  - Palate
    - Petechiae?
  - Nose
    - Congestion or rhinorrhea? (suggesting viral infection rather than strep)
- Lymphadenopathy
  - Superficial cervical chain (commonly enlarged with strep)
  - Submandibular
  - Submental
  - Occipital
- Skin
  - Scarlatiniform/sandpaper rash (arms, legs, trunk)
- MSK: ROM of neck
  - Neck extension/flexion (eyes to the sky, chin to chest motion)
    - Pain/limitations with extension could suggest retropharyngeal abscess
  - Neck lateral flexion (ear to shoulder motion)
  - Neck lateral rotation (looking over shoulder as if saying ‘no’)
    - Pain/limitation with lateral flexion/rotation could suggest a peritonsillar abscess! (pain/limitation would be unilateral to infection site)
- Abdomen (important as patients with strep may have abdominal pain)
  - Inspection
  - Auscultation
  - Palpation (superficial and deep)
    - Any splenomegaly to suggest mononucleosis as the cause of sore throat?

**Next, review assessment rubric with students and demonstrate the throat swab according to the steps below:**

1. Instruct patient (student) to sit erect, facing provider (you).

- Point out that children may not be cooperative, so will likely need to recruit extra hands to help (parents/guardians are ideal). Child can sit on exam table (ideal) or in parent’s/guardian’s lap. Parent/guardian may need to hold patient’s arms down.

2. Instruct patient to tilt head back.

3. Ask patient to open mouth and say “ah” when starting swab.

-If developmentally appropriate, may ask the child to “pant like a puppy.”

4. Insert swab without touching lips, teeth, tongue, cheeks, uvula.

5. Gently, but quicky, swab BOTH tonsils and pharynx from side to side, contacting any inflamed or purulent sites.

6. Carefully withdraw swab without touching oral structures (this will contaminate the test!).

**For remainder of the session, encourage students to practice the exam/procedure on each other and/or ask questions regarding what they have learned.**

**POST-SKILLS ASSESSMENT & BEDSIDE TEACHING SESSION:**

**Materials required:**
- Gloves

- Tongue depressors

- Throat swabs

- Light source (may use phone if otoscopes unavailable)

**Attendees:**

- Clerkship students on pediatric rotations that allow for afternoon activities (such as hospital medicine, elective, nursery)

-At least 1 facilitator

**Pre-session planning required:**Identify 1-2 patients in the hospital who agree to be examined by you and medical students. Ideally this patient would have a head/neck complaint relevant to the content of this session, but it is not required, as normal findings can also be helpful to recognize!

**You should allow for 1 hour to complete the skills/bedside teaching session later in the clerkship. Ideally, these will be offered throughout the clerkship to promote small group learning and the ideal student:teacher ratio.**

This session will begin with a clinical skills (focused physical exam and throat swab skills) assessment. One-by-one, students should assume the role of “doctor” and “patient”. Students will be called up two at a time, ideally in a location away from the rest of the group. You as the facilitator will be an observer. You will prompt the student doctor to “perform a focused physical exam on a patient presenting with sore throat and then perform a throat swab” and instruct them to verbalize what they are seeing (or not seeing) on exam. Of note, it is best if students rotate through the roles one-by-one rather than in pairs, as to avoid one student copying another. You as the facilitator will fill out the assessment rubric (Appendix F) while NOT providing any feedback or prompting. Each student should be allowed 2 minutes to complete the task.

After each student has completed the task, give feedback to the group/individuals regarding what they did well and suggestions for improvement. Review the assessment rubric together.

For the remainder of the hour, you will walk students through the examination skills on a hospitalized patient using the checklist (Appendix C), ideally with a head/neck complaint (but acceptable if not available). Our recommended approach is to demonstrate first, and then allow students to practice individually, while you give them real-time feedback.

**KEY TO KNOWLEDGE QUESTIONS:**

**1. A**

**2. D**

**3. E**

**4. B**

**5. TRUE**
