# Supplementary material for: A Hypothesis-Driven, Near-Peer Physical Diagnosis Module on Streptococcal Pharyngitis Within the Pediatrics Clerkship
Source: MedEdPORTAL. 2024 Oct 4;20:11448. doi: 10.15766/mep_2374-8265.11448 (PMC11450068; doi:10.15766/mep_2374-8265.11448)
Supplement: Supplementary file 1 — Physical Diagnosis Streptococcal Pharyngitis.pptxFacilitator Guide.docxSore Throat Physical Exam Bedside Checklist.docxPremodule Survey.docxPostmodule Survey.docxThroat Swab Skills Assessment Rubric.docx [file mep_2374-8265.11448-s001.zip › C. Sore Throat Physical Exam Bedside Checklist.docx]

**Sore Throat Physical Exam Bedside Checklist**

PHYSICAL EXAM:

- HEENT:
  - Tonsils
    - Presence or absence (s/p T&A?)
    - Size (2+, 3+, 4+)
    - Color (Erythema?)
    - Exudates
  - Uvula
    - Deviated vs midline
    - Color
    - Petechiae
  - Palate (hard vs soft)
    - Petechiae
  - Nose
    - Congestion or rhinorrhea (suggesting viral infection)
- Lymphadenopathy
  - Superficial cervical chain
  - Submandibular
  - Submental
  - Occipital
- Skin
  - Scarlatiniform/sandpaper rash (arms, legs, trunk)
- MSK: Neck
  - ROM (pain w/ extension could suggest retropharyngeal abscess)
- Abdomen (patients with Strep may have abdominal pain)
  - Inspection
  - Auscultation
  - Palpation (superficial and deep)
    - Any splenomegaly to suggest mononucleosis as the cause of sore throat?
