# Supplementary material for: A Hypothesis-Driven, Near-Peer Physical Diagnosis Module on Streptococcal Pharyngitis Within the Pediatrics Clerkship
Source: MedEdPORTAL. 2024 Oct 4;20:11448. doi: 10.15766/mep_2374-8265.11448 (PMC11450068; doi:10.15766/mep_2374-8265.11448)
Supplement: Supplementary file 1 — Physical Diagnosis Streptococcal Pharyngitis.pptxFacilitator Guide.docxSore Throat Physical Exam Bedside Checklist.docxPremodule Survey.docxPostmodule Survey.docxThroat Swab Skills Assessment Rubric.docx [file mep_2374-8265.11448-s001.zip › D. Premodule Survey.docx]

**Appendix D: Physical Diagnosis: Streptococcal Pharyngitis | Premodule Survey**

**Please complete the survey below.**

**Thank you!**

**During what block did you have your pediatrics clerkship?**

- **Block 1**
- **Block 2**
- **Block 3**
- **Block 4**
- **Block 5**

**Confidence Quiz**

**How confident are you in your understanding of the role of antibiotics in treating streptococcal pharyngitis?**

- **Not at all confident**
- **Slightly confident**
- **Somewhat confident**
- **Quite confident**
- **Extremely confident**

**How confident are you in your ability to use modified Centor criteria to stratify patients into risk categories to determine whether they should be tested/treated for streptococcal pharyngitis?**

- **Not at all confident**
- **Slightly confident**
- **Somewhat confident**
- **Quite confident**
- **Extremely confident**

**How confident are you in your knowledge of the most specific physical examination findings of streptococcal pharyngitis?**

- **Not at all confident**
- **Slightly confident**
- **Somewhat confident**
- **Quite confident**
- **Extremely confident**

**How confident are you in your ability to perform a focused physical exam related to the chief complaint of throat pain? Not at all confident**

- **Slightly confident**
- **Somewhat confident**
- **Quite confident**
- **Extremely confident**

**How confident are you in your ability to perform a throat swab accurately in a pediatric patient?**

- **Not at all confident**
- **Slightly confident**
- **Somewhat confident**
- **Quite confident**
- **Extremely confident**

**Knowledge Quiz**

**1. A 10-year-old male presents to the clinic with a 1-day history of sore throat. On arrival, his vitals are as follows; T 38.4C, HR 100, BP 100/60, RR 14, O2 sat 100% on room air. He denies cough, congestion, fever, and rhinorrhea. Mom states that his 7-year-old sister had similar symptoms 8 days ago. On exam, you note a tender, enlarged cervical lymph node on the left and erythematous tonsils with small areas of white exudate bilaterally. What is this patient’s modified Centor score? What is the best next step in management?**

**A. Score of 5: Perform rapid antigen detection test and send confirmatory culture.**

**B. Score of 4: Perform rapid antigen detection test. If negative, do not send culture and do not treat with antibiotics.**

**C. Score of 3: Defer diagnostic testing and encourage supportive care only as this patient likely has viral pharyngitis.**

**D. Score of 5: Defer rapid antigen detection test and only send a culture, as this is the gold standard for streptococcal pharyngitis.**

**2. Treating streptococcal pharyngitis with antibiotics can help prevent which post-infectious complication(s)?**

**A. Post-streptococcal glomerulonephritis (PSGN)**

**B. Acute rheumatic fever**

**C. Peritonsillar abscess**

**D. B and C**

**E. A and B**

**3. What is (are) the most specific physical examination finding(s) of streptococcal pharyngitis?**

**A. Palatine petechiae**

**B. Tonsillar exudates**

**C. Scarlatiniform rash**

**D. A and B**

**E. A and C**

**4. Choose the answer that represents the most accurate way to perform a throat culture.**

**A. Instruct patient to lay supine on exam table, open mouth, say “ah,” swab one side of tonsil avoiding any exudative areas, remove without touching oral structures.**

**B. Instruct patient to tilt head back, say “ah,” depress anterior third of tongue, swab tonsillar area from side-to-side, remove without touching oral structures.**

**C. Instruct patient to lean forward, open mouth, depress anterior third of the tongue, swab posterior soft palate, remove without touching oral structures.**

**D. Instruct patient to tilt head back, say “ah,” depress tongue with blade (staying in place if patient gags to maintain positioning), swab uvula and tonsils, remove without touching oral structures.**

**5. No single element of a patient’s history or physical examination is sufficient to exclude or diagnose strep throat.**

**A. True**

**B. False**
