# Supplementary material for: A Hypothesis-Driven, Near-Peer Physical Diagnosis Module on Streptococcal Pharyngitis Within the Pediatrics Clerkship
Source: MedEdPORTAL. 2024 Oct 4;20:11448. doi: 10.15766/mep_2374-8265.11448 (PMC11450068; doi:10.15766/mep_2374-8265.11448)
Supplement: Supplementary file 1 — Physical Diagnosis Streptococcal Pharyngitis.pptxFacilitator Guide.docxSore Throat Physical Exam Bedside Checklist.docxPremodule Survey.docxPostmodule Survey.docxThroat Swab Skills Assessment Rubric.docx [file mep_2374-8265.11448-s001.zip › F. Throat Swab Skills Assessment Rubric.docx]

| **SKILL/STEP** | **SATISFACTORY** | **UNSATISFACTORY** | **NOT PERFORMED** |
| --- | --- | --- | --- |

| Mouth/pharynx exam **(prompt student to name the anatomical structures they are assessing):** Using a light, examined pharynx/tonsils, palate, uvula | *must name/examine ALL using a light* | *named/examined SOME but not all / did not use light* |  |
| --- | --- | --- | --- |
| Using a light, examined nose for congestion/rhinorrhea | *using light, examining both sides* | *did not use light, only looked at one side* |  |
| Palpated for cervical adenopathy | *both sides of neck* | *one side of neck* |  |
| Assessed ROM (range of motion) of neck (flexion, extension, left/right lateral rotation, left/right lateral flexion) | *assessed all directions* | *assessed some but not all directions* |  |
| Assessed patient for signs of rash | *must at least examine arms + one other area (can verbalize, do not expose classmates!)* | *did not assess more than one location* |  |
| When it was time to swab, instructed the patient to sit erect, facing the provider. If the patient is uncooperative, recruits help with stabilizing head/extremities |  |  |  |
| Instructed patient to tilt head back |  |  |  |
| Asked patient to open mouth and say “ah” when starting swab |  |  |  |
| Depressed anterior third of tongue with blade |  |  |  |
| Inserted swab without touching lips, teeth, tongue, cheeks, uvula |  |  |  |
| Gently, but quickly swabbed both tonsils and pharynx from side-to-side, contacting any inflamed or purulent sites |  |  |  |
| Carefully withdrew swab without touching oral structures |  |  |  |
